# A semi-automatic seed point-based method for separation of individual vertebrae in 3D surface meshes: a proof of principle study

**DOI:** 10.1007/s11548-021-02407-z

**Published:** 2021-05-27

**Authors:** Peter A. J. Pijpker, Tim S. Oosterhuis, Max J. H. Witjes, Chris Faber, Peter M. A. van Ooijen, Jiří Kosinka, Jos M. A. Kuijlen, Rob J. M. Groen, Joep Kraeima

**Affiliations:** 1 3D-Lab and Department of Neurosurgery, University of Groningen, University Medical Center Groningen, Hanzeplein 1, 9713 GZ Groningen, The Netherlands; 2 3D-Lab and Bernoulli Institute, University of Groningen, University Medical Center Groningen, Hanzeplein 1, 9713 GZ Groningen, The Netherlands; 3 3D-Lab and Department of Oral and Maxillofacial Surgery, University of Groningen, University Medical Center Groningen, Groningen, The Netherlands; 4 Department of Orthopedic Surgery, University of Groningen, University Medical Center Groningen, Groningen, The Netherlands; 5 Department of Radiation Oncology and Data Science Center in Health, University of Groningen, University Medical Center Groningen, Groningen, The Netherlands; 6 Bernoulli Institute, University of Groningen, Groningen, The Netherlands; 7 Department of Neurosurgery, University of Groningen, University Medical Center Groningen, Groningen, The Netherlands

**Keywords:** Spine, Vertebra, Segmentation, Virtual surgical planning (VSP), Computed tomography, Seed point segmentation, 3D surface segmentation

## Abstract

**Purpose:**

The purpose of this paper is to present and validate a new semi-automated 3D surface mesh segmentation approach that optimizes the laborious individual human vertebrae separation in the spinal virtual surgical planning workflow and make a direct accuracy and segmentation time comparison with current standard segmentation method.

**Methods:**

The proposed semi-automatic method uses the 3D bone surface derived from CT image data for seed point-based 3D mesh partitioning. The accuracy of the proposed method was evaluated on a representative patient dataset. In addition, the influence of the number of used seed points was studied. The investigators analyzed whether there was a reduction in segmentation time when compared to manual segmentation. Surface-to-surface accuracy measurements were applied to assess the concordance with the manual segmentation.

**Results:**

The results demonstrated a statically significant reduction in segmentation time, while maintaining a high accuracy compared to the manual segmentation. A considerably smaller error was found when increasing the number of seed points. Anatomical regions that include articulating areas tend to show the highest errors, while the posterior laminar surface yielded an almost negligible error.

**Conclusion:**

A novel seed point initiated surface based segmentation method for the laborious individual human vertebrae separation was presented. This proof-of-principle study demonstrated the accuracy of the proposed method on a clinical CT image dataset and its feasibility for spinal virtual surgical planning applications.

## Introduction

Patients suffering from spinal pathologies that cause instability of the spine are often treated with posterior rigid fixation surgery in order to immobilize the spine and to prevent further damage to the spinal cord [1–3]. Screws are inserted bilaterally through the pedicles or in the lateral mass at each segment level. The spinal segments to be fused are immobilized by the insertion of a rod through the polyaxial screw heads.

Accurate screw insertion is important to minimize the risk of injuring nearby vital structures, such as the spinal cord and the nerve roots, and to facilitate a biomechanical stable fixation [4]. Due to advances in medical technology and the well-understood importance of accurate screw insertion, three-dimensional (3D) virtual surgical planning (VSP) has become increasingly popular since its emergence approximately two decades ago [5–10]. In the University Medical Center Groningen, VSP including the use of 3D printed drill guides is one of standard techniques for pedicle or lateral mass screw insertion in the cervical and upper thoracic spine.

A crucial part of the spinal VSP workflow is bone segmentation in order to obtain 3D bone models from the CT image data. Bone segmentation is the process of partitioning bone tissue from surrounding soft tissues in medical image data. A patient-specific modeling workflow necessitates segmentation of all individual human vertebrae for precise preoperative planning of screw trajectories. However, the complex shape and irregularities in bone density make the delineation of individual vertebrae challenging. Moreover, the per level varying signal-to-noise ratios and the occurrence of CT artifacts further hinder segmentation [11, 12]. In order to obtain accurate bone models, the current workflow requires substantial manual image pre-processing by specially trained medical engineers, which is time consuming and impedes the cost-effectiveness of VSP.

Development of (semi-)automated image processing has led to new segmentation methods based on statistical shape models and atlases [13–17]. Since these methods have been trained on available databases the algorithm generally underperforms in situations where the anatomy differs substantially from the average spine, i.e., cases with severe spinal deformation. In fact, surface errors are reported to be as high as 5.36 mm [20]. The resulting erroneous models are simply not suitable for computer-aided design and might lead to serious misfit of instruments. The more recent evolution of machine learning techniques reportedly yields smaller errors and might therefore be more useful for computer-aided design of surgical instruments, but these techniques are not yet implemented in commercially available software and should still be evaluated to be sufficiently reliable [18]. Threshold-based bone segmentation is, therefore, still considered the gold standard for VSP applications. The method is reliable since it relies on Hounsfield Units calibrated with reference to water. Moreover, it is not influenced by exceptional changes in anatomy resulting from severe spinal deformation pathologies.

Since image segmentation is one of the most crucial but time-consuming tasks in spinal VSP, the workflow should be optimized by introducing new methods for separating the individual vertebrae. In order to meet the clinical demand, a more automated approach for splitting of individual vertebrae that relies on the proven reliability and validity of threshold-based segmentation is desired. To address this issue, we propose a semi-automated software tool that uses threshold-based 3D bone surface mesh derived from CT image data and separates individual vertebrae based on 3D surface positioned seed points. The proposed method’s efficacy and the relation to the number of used seed points were evaluated on a patient dataset. An accuracy and time comparison was made between the proposed method and the current standard manual segmentation method.

## Methods and materials

### The proposed algorithm

The seed point-based method presented in this paper can best be characterized as a surface seeding method, because it is a seed point-based surface segmentation method applied to the bone surface as opposed to direct segmentation of the CT-scan volume. Before this surface seeding method can be applied, a 3D bone surface mesh of the human spine needs to be extracted from the CT image data. For the purpose of this study, global thresholding and marching cubes triangulation were used, which is currently the standard in VSP.

#### Surface region-growing

**Fig. 1:**
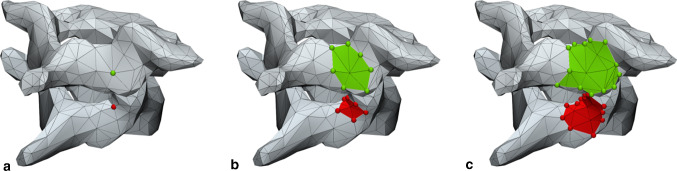
Conceptual image of two steps of the iterative surface region-growing based on normals between vertices. **a** Before the region-growing, seed points (shown in green and red) for different bone segments are placed. **b** In the first iteration, vertices neighboring the seed points are assigned their respective regions if the angle between their normals is low enough. **c** In the second iteration, vertices bordering the newly assigned vertices can be added to the segments, and so on until there are no newly assigned vertices. Note that not all of the neighboring vertices are assigned to the two segments, because sharp edges are reached

Seed points are manually placed on all parts of the extracted bone surface by hand. At least one seed point is required for each bone segment. The method, however, allows to place a higher number of seed points per segment. After the initial seed point placement, the region-growing algorithm can be started. The regions are grown from the seed points using an algorithm with a breadth-first approach. At first, the seed point vertices are added to a last-in-first-out queue. As long as the queue is not empty, the vertex at the front of the queue is dequeued. Each of its neighboring vertices is added to the back of the queue and labeled with the region of the current vertex, provided that they do not have region label yet and are also ‘connected’ to the current vertex. Two neighboring vertices are ‘connected’ when the angle between the vertex normals does not exceed a maximum allowed threshold angle. This maximum allowed threshold angle is a parameter which is initially set by the user. A pseudocode depiction of the region-growing algorithm can be seen in Algorithm 1. The connectivity constraint causes vertices in smooth areas to form connected surfaces through which the region-growing is propagated. On the other hands, larger angles between normals of neighboring vertices which exceed the threshold angle form natural barriers. The effect can be compared to that of an oil spill, spreading circumferentially over the smooth regions of the surface, starting from the seed points (Fig. 1).
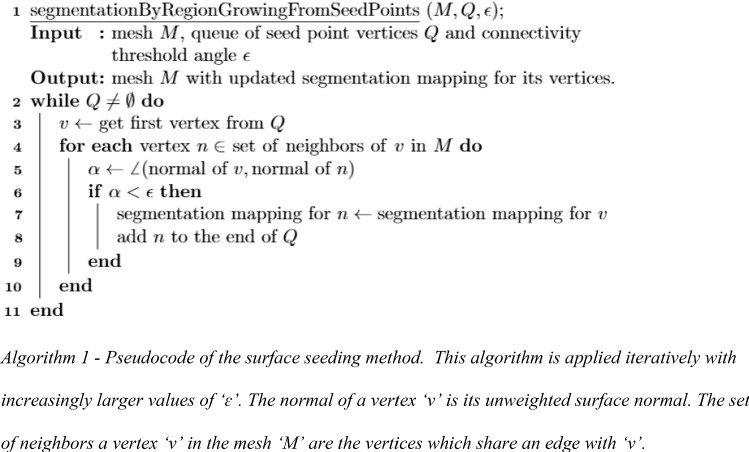

#### Iterative threshold adaptation

The threshold angle for connectivity is set to be low initially. After an initial region-growing pass, the user may wish to increase the connectivity threshold by increasing the maximum allowed angle between vertex normals. A new region-growing algorithm pass is then initiated. Between region-growing algorithm iterations, the user can undo the last region-growing pass and add additional seed points. This interactive process of automatic region-growing steps and possible user intervention can be repeated until all vertices are properly assigned to spinal segments.

#### Surface segmentation application

The surface seeding segmentation algorithm described in this paper was implemented as a C+ + application for the purpose of 3D VSP. The input of the seed point algorithm is the 3D bone surface mesh derived from CT image data. In the application, the mesh is obtained by global thresholding and triangulated by marching cubes, equivalent to the current standard in VSP. The user interaction of the segmentation algorithm takes place through a graphical user interface (GUI), which includes a 3D projection of the bone surface to be segmented as well as an options menu. Seed points can be placed on the projected bone surface by mouse clicks.

### Evaluation

#### Clinical datasets

CT image datasets from five human patients who previously underwent spinal fixation surgery by use of individualized 3D-printed drill templates were obtained from the University Medical Center Groningen. The datasets are representative for future clinical cases in terms of uniform spine scan and reconstruction protocol (0.6 mm slice thickness, 0.4 mm slice increment, sharp reconstruction kernel I70h). The cohort data range in age from 12 to 75.

#### Ground truth construction

To build a ground truth for the validation of our algorithm, all cervical and upper thoracic human vertebrae (when available in the dataset) were manually segmented by threshold segmentation and the split mask feature in Mimics v20 (Materialise, Leuven, Belgium) by an experienced clinician (PP) and checked by the surgeon (JKu). In addition, the time required for manual segmentation was measured, from the moment of loading the dataset until the export of all models.

#### Seed point-based surface segmentation

**Fig. 2:**
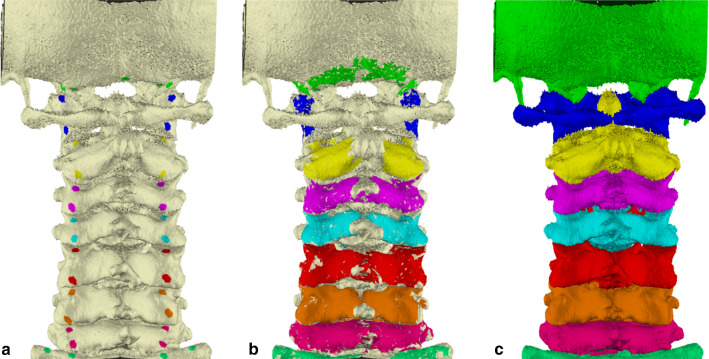
The results of applying the algorithm to the first dataset, showing, **a** the initially set seed points on each level, **b** the colored parts during surface region-growing after 3 iterations, and **c** the final result after finishing the iterative process

**Fig. 3:**
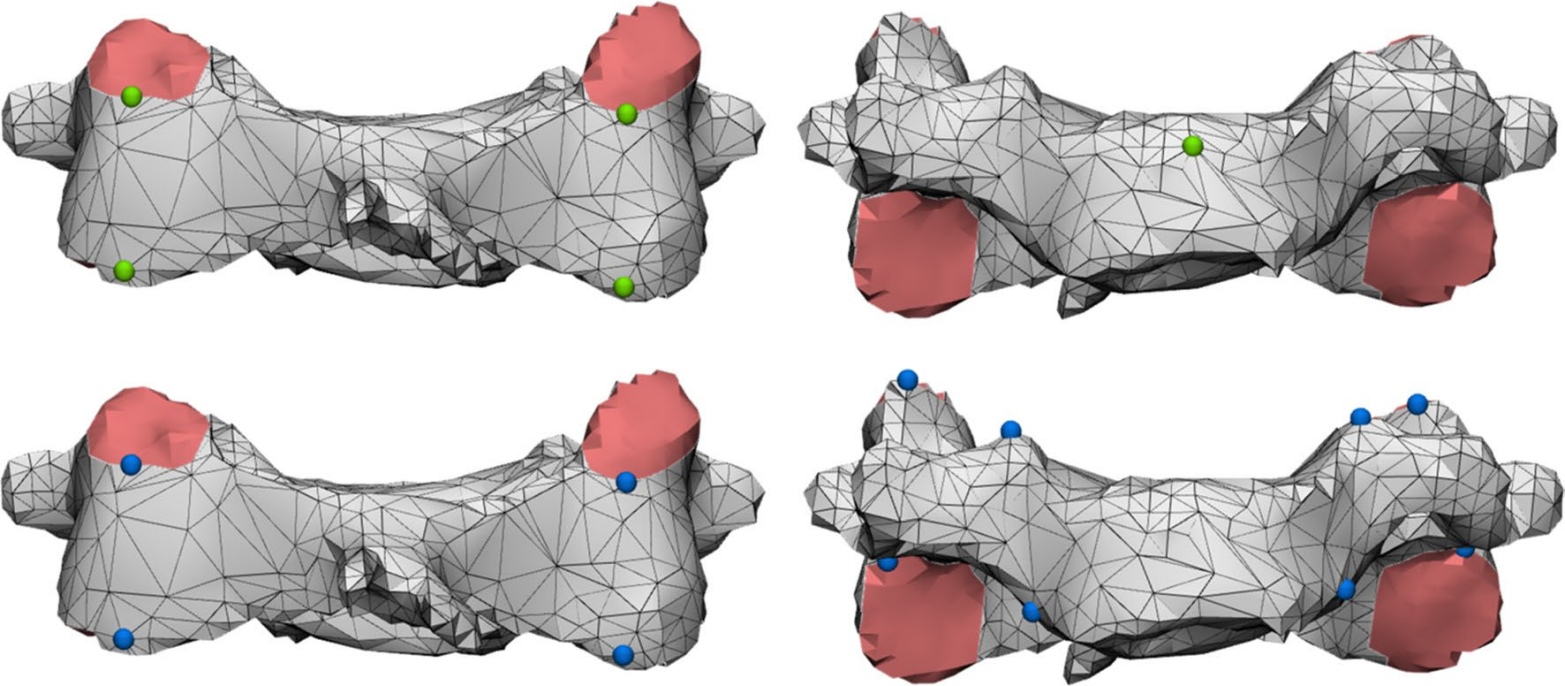
The predefined locations for the 5 seed points (green) and 12 seed points (blue) scenarios displayed on an already preprocessed and segmented example human vertebra, showing the vertebra in posterior view (left) and the anterior view (right). In case of using 5 seed points (green) per vertebrae, the posterior seed points were located on the edge of the superior and inferior articulating process (red) bilaterally. Anteriorly, one seed point was positioned on the center of the vertebral body. In case of 12 seed points (blue), additional seed points were positioned at the anterior articular area, and 4 seed points were positioned on the vertebral body, instead of one seedpoint

All individual vertebrae for each patient were segmented and reconstructed to 3D models using the presented method (Fig. 2). To investigate whether a higher number of seed points improves accuracy, the algorithm was applied twice to each dataset, once with 5 seed points per vertebra and once with 12 seed points. The seed points were predefined near anatomical locations that are likely to touch adjacent vertebrae. These anatomical locations are also easy to find in the 3D space, making the study outcomes reproducible (Fig. 3). Two observers performed the segmentations independently to determine the inter-observer variability. For each case, the 3D bone surface was derived from CT image data using the previously chosen HU threshold. The initial connectivity threshold angle was set to 5 degrees, and this was increased with 5 degrees after each iteration of region-growing.

#### Evaluation metrics

**Fig. 4:**
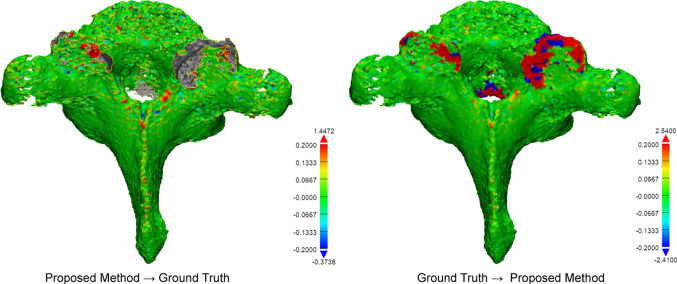
Example of surface-to-surface distance plot (case number 4, T2 vertebra, 12 seed points, observer 1). Here, the green represents a perfect segmentation, the red color a positive error, and the blue a negative error. The gray, yellow-outlined, areas indicate the holes in the surfaces. The color grading legend is displayed in millimeters. Note that the typical areas of error, like the articular processes, are colored in red

The algorithm performance was evaluated on both the whole vertebra and the vertebral substructures. Using 3-matic Research v12 (Materialise, Leuven, Belgium) a surface-to-surface Euclidean distance could be calculated from each surface point to the nearest point on the reference model (Fig. 4). The distance error was calculated from seed point model to ground truth and contrariwise (both-ways), in order to detect protrusions and missing parts. The list of analysis values was then exported to calculate relevant parameters that reflect the segmentation accuracy. The calculated parameters include the average symmetric surface distance (ASSD), the average asymmetric surface distance (AASD), and the root mean square of the surface distance, all according to the formulas provided by Heimann et al. [19] In addition, the maximum surface distance, the 95th percentile of error, and the percentage of surface with a distance error underneath the 0.2 mm were calculated.

By visual inspection, it was noticed that the performance of the algorithm varies at different vertebral segments. The vertebrae were therefore partitioned into anterior and posterior substructures by positioning a vertical cutting plane midway through the pedicles. Moreover, the laminae were separately analyzed after isolation using the wave brush marking tool.

All aforementioned parameters were calculated from the surface distance data using a custom-written Python script and presented as descriptive statistics. The inter-observer variability was supported by the calculation of the intraclass correlation coefficient (ICC). The proposed methods and manual segmentation times were compared by using a paired t-test. Data analysis was performed using IBM SPSS statistics version 23 (IBM corp., Armonk, NY, the USA).

## Results

**Fig. 5:**
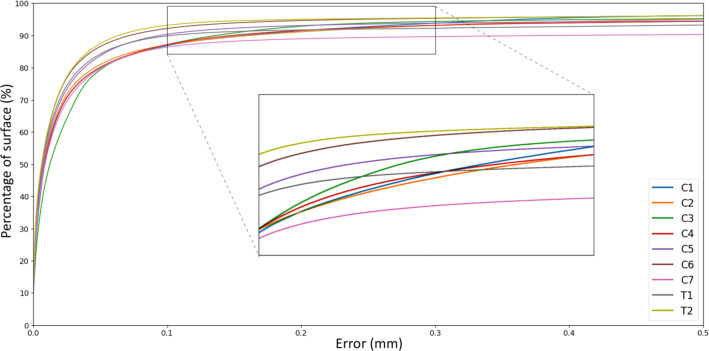
Cumulative error distribution plot between the proposed method and the ground truth differentiated by vertebral level in case of using 12 seed points per vertebra. The plot visualizes the maximum error and its variance between spinal levels at each chosen percentile of the total surface mesh

**Fig. 6:**
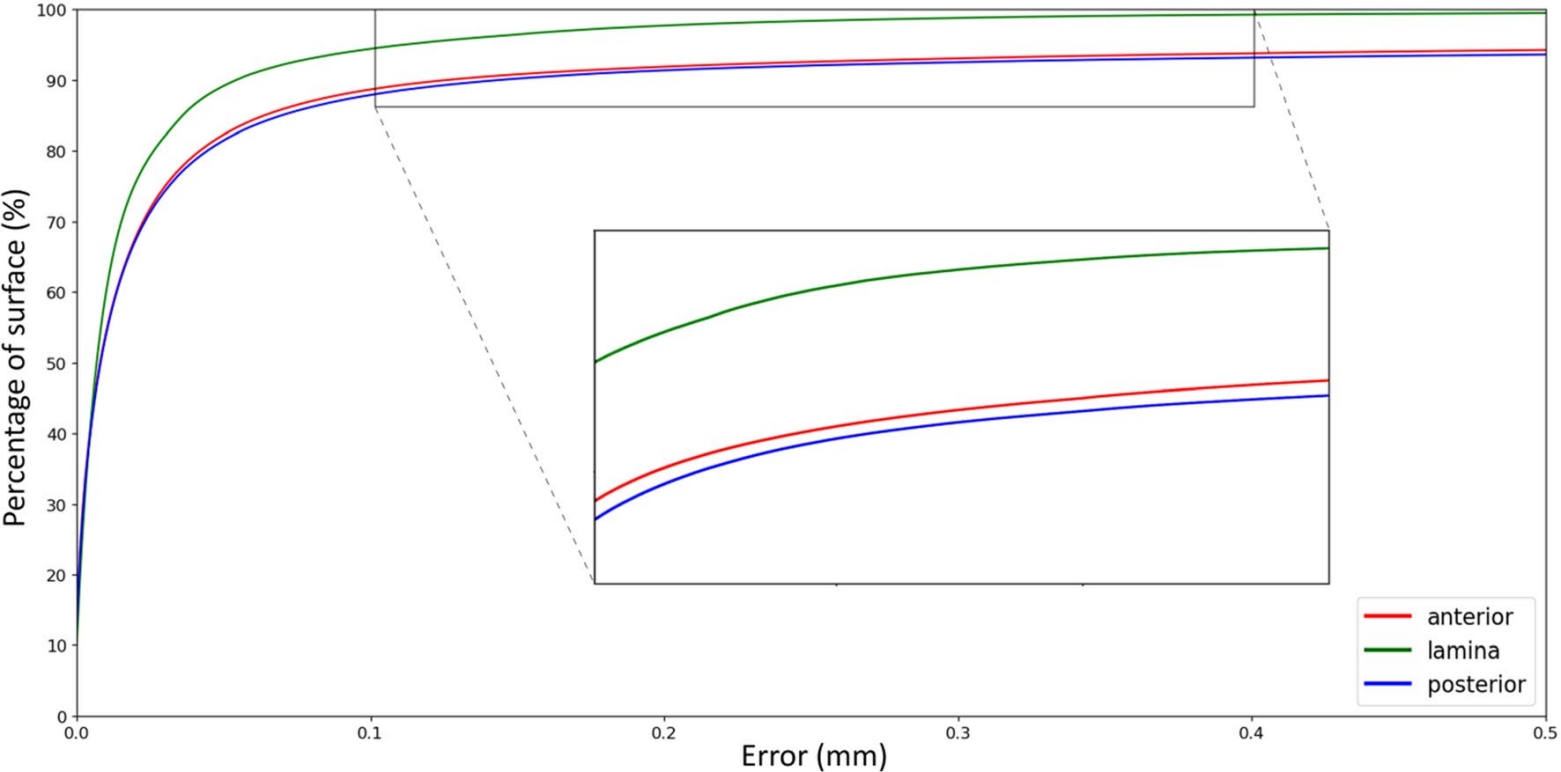
Cumulative error distribution plot for the anterior, posterior and lamina substructures in case of using 12 seed points per vertebra. The plot visualizes the maximum error and its variance between vertebral substructures at each chosen percentile of the total surface mesh

The results show a clear algorithm performance difference between the 5 and 12 seed point scenario (Table 1). The ASSD for using 5 seed points was 0.52 mm and decreased to 0.23 mm when using 12 seed points. The 95th percentile for 5 and 12 seed points was 3.07 mm and 0.69 mm, respectively. To put this in perspective, the width of a typical T1 vertebral body measures 26.5 mm [20]. The detailed segmentation accuracy results for the 12 seed point scenario are listed in Table 2, providing a distinction between cases, vertebral levels, and anatomical regions. Grouping per case showed that the ASSD ranges from 0.05 mm for Case 2 to 0.32 mm for Case 5, and the respective 95th percentiles were 0.09 mm and 1.47 mm. The ASSD for the different anatomical levels ranged from 0.11 mm in C1 vertebrae to 0.69 mm in C7 vertebrae. The cumulative error distribution plot provides insight in the distribution of error for each vertebral level and shows that for all levels except for the C7 vertebra, 90 percent of the surface demonstrates an error below 0.2 mm (Fig. 5). The results for the different anatomical regions show an ASSD of 0.25 mm for the anterior substructures and 0.49 mm for the posterior parts of the vertebrae. When limiting to the laminar region, the ASSD decreased to 0.03 mm. The respective 95th percentiles were found to be 0.99 mm (anterior), 0.71 mm (posterior), and 0.11 mm (lamina). The performance of the algorithm between the defined anatomical regions is illustrated in the cumulative error distribution plot (Fig. 6). An inter-observer correlation (ICC) of 0.96 between the two raters was found.

**Table 1:** A comparison between using 5 or 12 seed points per vertebra showing metrics based on surface distance measurements

	ASSD (mm)	AASD (mm)	RMS (mm)	Max error (mm)	95th pctl. (mm)	pct. error. < 0.2mm
5 seeds	0.52	0.37	2.20	36.05	3.07	88%
12 seeds	0.23	0.14	1.27	24.48	0.69	92%

*ASSD* average symmetric surface distance; *AASD* average asymmetric surface distance; *RMS*, root mean square of surface distance. Values are displayed in millimeters, except for the last column, which shows a percentage

**Table 2:** Accuracy results for the 12 seed point scenario, providing a distinction between the different cases, the vertebral levels, and the anatomical regions

		ASSD (mm)	AASD (mm)	RMS (mm)	Max error (mm)	95th pctl. (mm)	pct. error. < 0.2 mm
Case No.	Case 1	0.26	0.17	1.49	24.48	0.98	92%
	Case 2	0.05	0.01	0.43	10.95	0.09	98%
	Case 3	0.26	0.03	0.76	19.11	0.98	73%
	Case 4	0.16	0.09	1.09	21.90	0.14	96%
	Case 5	0.32	0.24	1.47	16.09	1.47	92%
Level	C1	0.11	− 0.01	0.49	11.38	0.36	91%
	C2	0.13	0.04	0.70	14.46	0.49	91%
	C3	0.17	0.08	0.89	19.11	0.43	93%
	C4	0.12	− 0.03	0.43	6.79	0.67	92%
	C5	0.21	0.13	1.13	16.09	0.68	93%
	C6	0.08	− 0.01	0.42	15.30	0.24	95%
	C7	0.69	0.63	2.69	24.48	5.90	89%
	T1	0.21	0.11	1.10	21.90	0.95	92%
	T2	0.08	0.04	0.41	8.60	0.20	95%
Region	Anterior	0.25	0.19	1.40	24.48	0.71	92%
	Lamina	0.03	0.01	0.19	12.54	0.11	98%
	Posterior	0.49	0.41	4.12	64.97	0.99	91%

The proposed method yielded consistently lower processing times compared to manual segmentation (Table 3). The manual method took 18.52 min per subject (all vertebrae) on average. When using the proposed seed point algorithm, segmentation times significantly reduced to 9.74 min per subject in case of the 12 seed point scenario (*P* = 0.021) and further reduced to 4.94 min in case of the 5 seed point scenario (*P* = 0.016).

**Table 3:** Time measurements, averaged over 2 raters, for segmentation using the proposed seed point method compared to using the manual segmentation method

	Proposed method (min)		Manual method(min)
	5 seeds	12 seeds	
Case 1	9.08	19.35	34.57
Case 2	2.85	4.80	9.43
Case 3	4.28	7.52	19.97
Case 4	4.43	10.30	12.58
Case 5	4.05	6.73	16.07
Average	4.94	9.74	18.52
*P* value*	0.016	0.021	

*P value between the newly proposed method and the manual method.

## Discussion

The use of VSP in spinal surgery provides surgeons with a tool to precisely plan pedicle screw positions and use 3D-printed patient-specific guides to translate the plan to the operating theater. The current VSP workflow requires specific 3D expertise and is time consuming with regard to segmentation of the individual anatomical structures [21]. For the spine specifically, individual vertebrae labeling requires time-consuming manual image processing and therefore strongly influences the cost-effectiveness of VSP. This study presents a semi-automated user-independent method for individual vertebrae separation. It ultimately resulted in a significant time reduction and accurate 3D surface mesh segmentation of the individual vertebrae.

In VSP, the initial construction of accurate 3D bone models derived from CT image data is crucial, as all other steps in the VSP process heavily depend on the accuracy of these models. Although current software includes algorithms to speed up individual vertebrae labeling, it still is a laborious manual process. Optimizing the process of individual vertebrae segmentation can be roughly divided into two distinct approaches; (1) introducing new state-of-the-art algorithms that form individual vertebrae from the CT volume data (volume segmentation), or as proposed in this study, (2) using proven volume segmentation techniques and introduce subsequent surface partitioning methods for separating adjacent vertebrae.

1. Several novel volume segmentation approaches have been proposed in the literature, including statistical shape models, atlas-based methods, or more recently, machine learning-based methods [13–17]. The essence of fully automated segmentation methods is often based on time saving and the reduction of errors during the clinical interpretation of image data. Although the methods have the potential to be applied to a large number of datasets without manual user input, using the atlas- and statistical shape-based methods for VSP applications would currently be imprudent considering the reported average surface errors ranging from 0.64 mm up to 5.36 mm in an osteoporotic cohort [22]. On the other hands, the evolution of machine learning techniques, such as the recently presented fully convolutional network by Lessmann et al., leads to significant progress in the accuracy of automated vertebra segmentation [18]. To the best of our knowledge, such machine learning-based methods are, however, currently not implemented in commercially available software packages. Manual threshold-based segmentations are therefore still considered as standard method, also because they incorporate expert knowledge and can handle exceptional changes in anatomy resulting from severe spinal deformation pathologies.
2. In case of a mesh-based approach, as proposed in this study, segmentation is applied after obtaining a 3D surface model from the image data. In this context, we developed an algorithm that skips the laborious manual image post-processing and introduces a seed point-based surface segmentation method that is applied after surface triangulation. The GUI allows the user to interactively position seed points in 3D. An iterative region-growing process based on surface normals is then initiated which adds neighboring vertices to segments when the angle does not exceed the threshold angle. The GUI enables additional user interaction during the iterative thresholding, providing the ability to add seed points as required.

The results of this study have demonstrated highly accurate separation of individual vertebrae. The robustness was shown by applying the proposed method to a representative dataset including osteoporotic cases. Also, an excellent inter-observer correlation was found between both raters. The hypothesized relation between the number of seed points and segmentation accuracy was supported by an over 50% reduction of the ASSD in case of using 12 seed points per level compared to using 5 seed points. Based on the comparable distribution of error in Fig. 6, it could be concluded that the accuracy for anterior and posterior regions did not differ largely. The presupposed lower error in laminar region was confirmed by an ASSD of 0.03 mm, an explainable effect due to exclusion of surface areas that connect with surrounding regions and tend to introduce the highest errors. The laminar error is clinically negligible, as against to a reported accuracy up to 0.62 mm for global thresholding alone [23]. Upon evaluation of the specific anatomical regions, an ASSD of 0.69 mm was found for the C7 vertebra. The often inferior image quality at the cervicothoracic junction, mostly due to shoulder artifacts, probably is an important source of error. Moreover, the ribs at the cervicothoracic junction might have been falsely assigned to the C7 vertebra and introduce severe surface errors. CT image noise is another commonly mentioned source of error, however, since CT noise is present throughout the whole scan and the error in the lamina was almost negligible, we suspect this is the least important source of error. Though, it has to be said that the noise-related error might also be present in ground truth. Compared to the current manual segmentation method, the algorithm proved to be significantly faster for both the 5 seed point scenario and 12 seed point scenario. The substantially higher segmentation time for the first case can be explained by the presence of severe degeneration in the facet joints. It has to be mentioned that the segmentation by current standard software was performed by an experienced rater, already passed the complete learning curve. This was not possible for the proposed method, because it is yet not part of clinical routine.

**Fig. 7:**
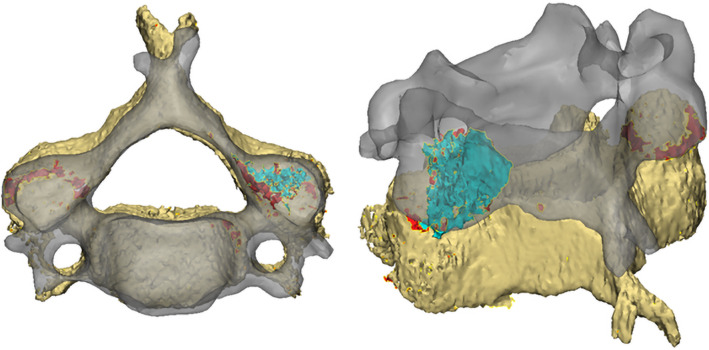
An example of a region-growing artifact which can arise with the surface seeding method. The meshes of the vertebrae contain holes where their facet joints touch, which causes the inferior facet joint surface (cyan) of the C5 vertebra (gray) to be wrongfully attributed to the C6 vertebra (beige). Note that the holes in the vertebrae meshes are shown in red

Despite the low average surface distances, it should be noted a relatively high maximum surface distance exists between the model created using mimics and the model created using the novel surface seeding algorithm. The reason for this lies in the fact that the extracted model contains an inner cortical surface that is formed by the border between the cortical bone and trabecular bone. The outliers in surface distance are the results of region-growing artifacts which arise when parts of the vertebra’s inner surface are added to an adjacent vertebra, as shown in Fig. 7. Currently, seeds cannot be placed on the inner surface, neither can they be placed at the articular parts of the facet joint.

In the literature, not much attention has been paid to surface segmentation techniques for clinical applications. Therefore, it is difficult to compare the results of the current study to earlier presented studies. Although the seed point method yielded excellent results, it is semi-automatic, and therefore different to most alternative approaches. Kim et al. presented a fully automatic surface-based method using 3D deformable fences [24]. The fences are generated based on valley information in the CT volume data. The 3D fences can be used for separating adjacent vertebrae from 3D surface models, and it can therefore be considered a surface segmentation method. Results were subjectively evaluated by a classification system, and therefore not comparable to our results. Lui et al. presented a surface-based method by means of spectral clustering. It was proven to be successful for small (50–4000 faces), preferably smooth, meshes with segment boundaries in concave regions [25]. This approach was not tested for clinical applications, but could potentially be interesting for bone segmentation, because the places where bone structures touch tend to form deep concavities. A so-called ‘intelligent scissoring’ or ‘randomized cuts algorithm’ was showed to be feasible for segmentation hand bones and might be useful for surface-based vertebra segmentation [26, 27]. However, for spinal application, the use might be complicated because the vertebra themselves also contain a large number of concavities. Moreover, surfaces extracted from high detail CT scans (up to 5.000.000 faces) may be challenging on conventional hardware.

The surface seeding segmentation program used in this study is in a prototype stage. There are a number of additions to this prototype application that are currently under development to further increase its clinical efficacy. Firstly, the prototype application only has a 3D view of the extracted bone surface and segments. A series of 2D overviews of the original CT images (axial, coronal, and sagittal) with the surface and bone outlined could provide valuable additional feedback. Another missing feature in the prototype application a hole-filling operation. Currently, the segments are exported as they are generated by the algorithm, including any holes that appear at the scan borders and where facet joints touch. However, for the VSP, solid body meshes are required. Artifacts like the one shown in Fig. 7 can potentially also be automatically trimmed during automatic hole filling, insofar as they are enclosed by the adjacent segment. It is also the absence of this closing holes operation that hindered the evaluation of the proposed method using the commonly used Dice coefficient (DC). Since the DC requires volumes rather than surfaces, the DC was not calculated. The current study was therefore limited in scope to surface distance-based measurements.

In this preliminary proof of principle study conclusions are based on a rather small dataset, which may be inadequate to draw definitive conclusions in terms of robustness in daily clinical practice. Nevertheless, it demonstrates the feasibility and serves as the basis for further development toward a clinically embedded 3D VSP tool. Minor variations between cases and vertebra were observed, but differences were minor and ASSD remained sub-millimeter. After implementation of additional features, which will be part of future work, the method should be evaluated on larger datasets to prove its robustness in clinical practice.

The results showed in this study have several surgical and VSP implications. It was found that the time required for individual vertebra segmentation can be considerably reduced when using the semi-automatic segmentation approach. Meanwhile, the segmentation accuracy remained high according to the percentage of surface with an error below 0.2 mm. This metric was set as clinically relevant cut-off value since patient-specific guides in our center are always designed with a 0.2 mm surface offset to cope with remaining soft tissue or small segmentation errors. Although artifacts were found, they do not impact the clinical efficacy of the surface seeding algorithm, because patient-specific drill guides are positioned on the outer surface. This is also reflected in the negligible error that was found when limiting the analysis to the laminar region. In this study, we chose to assess the use of 5 and 12 seed points for reasons of study simplicity and clarity and due to spinal anatomical features. In clinical practice, however, the number of seed points needed per vertebra is likely to vary between cases. The number of seed points should be judged in a clinical setting on a case-to-case basis, depending on bone quality, facet joint osteoarthritis, and degeneration of the intervertebral disks. In addition to this, the expert users have a substantial degree of control during the iterative process due to the ability to add extra seed points after each region-growing iteration.

In conclusion, we have described a semi-automated surface segmentation algorithm for individual vertebrae separation based on manually positioned seed points. The method relies on global threshold segmentation and introduces a seed point-based 3D mesh partitioning method for separating adjacent vertebrae. This proof-of-principle study demonstrated the accuracy of the proposed method on a clinical CT image dataset and the feasibility for spinal VSP applications.

## Data Availability

The datasets generated during and/or analyzed during the current study are available from the corresponding author on reasonable request.

## Code availability

The developed application and source code have been made publicly available for research purposes at https://github.com/TimOosterhuis/semiautomatic_CT_3D_surface_mesh_segmentation.
